# From Bottle Caps to Frisbee—A Case Study on Mechanical Recycling of Plastic Waste towards a Circular Economy

**DOI:** 10.3390/polym15122685

**Published:** 2023-06-14

**Authors:** Mohamad Hassan Akhras, Paul J. Freudenthaler, Klaus Straka, Joerg Fischer

**Affiliations:** 1 Competence Center CHASE GmbH, Hafenstraße 47-51, 4020 Linz, Austria; 2 Institute of Polymeric Materials and Testing, Johannes Kepler University, Altenberger Straße 69, 4040 Linz, Austria; paul.freudenthaler@jku.at; 3 Institute for Polymer Injection Moulding and Process Automation, Johannes Kepler University, Altenberger Straße 69, 4040 Linz, Austria; klaus.straka@jku.at

**Keywords:** plastics recycling, mechanical recycling, high-density polyethylene, post-consumer waste, packaging waste, digital product passport, regranulation, recyclates

## Abstract

This study demonstrates an open-loop recycling process of a specific post-consumer plastic waste stream. The targeted input waste material was defined as high-density polyethylene beverage bottle caps. Two methods of waste collection, informal and formal, were employed. Thereafter, materials were hand-sorted, shredded, regranulated, and then injection-molded into a flying disc (i.e., frisbee) as a pilot product. To observe the potential changes in the material throughout the entire recycling process, eight different test methods including melt mass-flow rate (MFR), differential scanning calorimetry (DSC), and mechanical tests were carried out on the various material states. The study showed that the informal collection led to a relatively higher purity in the input stream, which also appeared to have a 23% lower MFR value compared to that of the formally collected materials. The DSC measurements revealed a cross-contamination by polypropylene, which clearly affected the properties of all investigated materials. The cross-contamination led to a slightly higher tensile modulus in the recyclate, while the Charpy notched impact strength declined after processing by approximately 15% and 8% compared to those of the informal and formal input materials, respectively. All materials and the processing data were documented and stored online as a practical implementation of a digital product passport as a potential digital traceability tool. Furthermore, the suitability of the resulting recyclate to be used in transport packaging applications was also investigated. It was found that a direct replacement of virgin materials for this specific application is not possible without proper material modification.

## 1. Introduction

In alignment with Europe’s Green Deal, a new edition of the European action plan for a transition to a circular economy (CE) was released in 2020 [1]. The plan outlines certain actions to be considered in terms of product design for circularity, sustainable consumption of products, and circularity in production processes. Recently, plastics in particular have become one of the highly debated materials due to their association to single-use products [2,3,4]. In 2021, the global production of plastics exceeded 390 million metric tons (Mt) [5], and this number is expected to soar to 460 Mt by 2030 [6]. Therefore, a comprehensive set of initiatives has been launched by the EU strategy to integrate the plastics sector in the CE [7]. Consequently, as part of a new framework for eco-design and sustainable products, a number of mandatory and voluntary product requirements were reinforced or introduced. Furthermore, the legislative initiative also endorses the potential use of digitalization tools to improve the traceability and transparent accessibility to product information through digital solutions, such as a digital product passport (DPP) [1]. A DPP is a comprehensive digital record that aims to collect and store information on a product’s lifecycle and make it accessible to the involved parties including the end-consumers throughout its entire value chain to create a better understanding of the interrelations between the materials, the processes, and the final product [8,9].

Amongst other materials, plastics have an excellent potential for achieving a high degree of circularity [10]. In general, the transition from the conventional linear economy into a CE in the plastics industry brings significant ecological advantages. These advantages are most evident in terms of resource conservation, waste reduction, curbing greenhouse gas emissions, and preventing the leakage of plastic waste into the environment [2,3,4]. Although recycling is not the sole component of CE, in the case of plastics, it represents the optimal solution to extend the material’s life cycle and reduce the dependency on new fossil-based plastics. In recent years, the plastics recycling industry has evolved substantially to emerge as one of the key forces driving the transition towards a sustainable CE [11,12]. However, numerous challenges in this sector still need to be properly addressed and tackled [13]. When addressing the transition to a CE for plastics, packaging applications are certainly of great importance [11,14]. This is due to the fact that packaging products, which are mostly designed for single-use purposes, represent the largest market of global plastic production [5]. Around 40% of the total market demand of plastics in Europe is driven by packaging applications, which are primarily dominated by polyethylene terephthalate (PET) and polyolefins (POs), such as polyethylene (PE) and polypropylene (PP) [5]. As a consequence, plastic packaging is responsible for one of the largest existing post-consumer waste groups [5,15]. The multi-component composition of plastic beverage bottles consisting of bottle body, bottle cap, and label makes them a good example that represents the complexity of post-consumer packaging waste. PET has emerged as the material of choice worldwide for water and soft drink bottles. This is due to the exceptional durability, the light weight, and the superior optical properties of this resin [16]. The bodies of PET bottles are nowadays readily recycled, as multiple recycling schemes for PET have been established. If the bottles are properly collected and sorted, it is even possible to mechanically recycle them in a closed-loop system, namely bottle to bottle [16]. Moreover, open-loop systems are also possible as the bottle can be recycled into thermoforming sheets (i.e., bottle to sheet) [17] or into textile fibers (i.e., bottle to fiber) that can be used for products such as footwear and bags [18]. Furthermore, literature provides examples that highlight some potential reusing and repurposing routes of beverage bottle caps. For instance, Oliveira et al. [19] proposed the utilization of plastic beverage bottle caps as core elements in aluminum–plastic sandwich structures. Another study by Irem et al. [20] examined the feasibility of using bottle caps as an alternative fuel source in comparison to low-quality lignite. However, both examples do not promote the principles of CE; thus, other applications must be considered. In contrast to the bottle body and despite the growing market size and their resulting post-consumer waste fraction [21], the recycling options for the other beverage bottle components still seem to be very limited. The scarcity of clean and sorted fractions of beverage bottle caps poses a significant challenge to effectively recycling them. If collected properly, a clean recyclable input stream of these materials can be obtained either at specific waste collection stations or as a rejected fraction in PET beverage bottle recycling plants. Such streams are commonly used as recycled content in specific injection-molded PE-HD transport packaging applications (e.g., beer crates). Moreover, caps are often discarded separately from the bottles, which increases their likelihood of entering mixed waste streams or being released into the environment, including landfills and oceans, hence drastically reducing their recycling potential [15,22]. For instance, in a beach cleanup tour of the coastline in the Netherlands conducted by the North Sea Foundation in 2016, over ten thousand plastic beverage bottle caps were collected [21]. To prevent the release of such waste into the environment and to enhance the recyclability of these materials, recent EU legislation, specifically Directive (EU) 2019/904 on single-use plastics, thoroughly addresses this issue. The Directive mandates specific product design requirements for caps and closures of beverage packaging. As outlined in Article 6 of the directive, caps and lids can only be placed on the market if they remain attached to the containers during the product’s use phase [23].

The objective of this paper is to present a case study that aimed to investigate the recycling potential of post-consumer beverage bottle caps. An additional goal is to track the waste materials throughout the recycling process and identify any changes that occurred due to processing. Furthermore, a practical implementation of a DPP was also demonstrated in a simplified form as a potential digital tool for documenting material changes and storing product and process data. Finally, the article also discusses the suitability of the recyclate produced within the study as a possible replacement of virgin materials for a predefined packaging application (i.e., transport packaging).

## 2. Materials and Methodology

### 2.1. Scope of the Case Study “From Bottle Cap to Frisbee”

The case study was carried out as a cooperation between the Linz Institute of Technology (LIT) Factory at Johannes Kepler University Linz (Linz, Austria), Engel Austria GmbH (Schwertberg, Austria), the Upper Austrian head agency for waste treatment LAVU “O.Ö. Landes-Abfallverwertungsunternehmen GmbH” (Wels, Austria), and the R-Cycle initiative (Troisdorf, Germany) [24]. One of the main objectives of this case study was to provide a practical implementation of digitalization tools through the utilization of the DPP concept for the data documentation and traceability of a defined waste stream throughout an open-loop recycling process. This included information about the material property profile after each processing step as well as the process data. This data collection makes it possible to visualize possible changes in the material properties due to processing. Additionally, and certainly as important as the previous objectives, the case study was an attempt to raise awareness of CE and sustainable use of plastics among young people. This was achieved by involving that segment of end-consumers in the collection step of a specific waste stream, which enabled them to observe the journey of the waste materials from disposal to the conversion into a new product. High-density polyethylene (PE-HD) beverage bottle caps were selected as the target waste stream for the present case study. The frisbee, on the other hand, was chosen to highlight the social impact of the project by demonstrating to the young participants how their waste can be transformed into a useful item. Despite its simple geometry and straightforward requirements, such a product can also resemble several rigid packaging applications, where certain requirements, such as processability, mechanical properties, durability, and dimensional stability, are of primary importance in comparison to other material characteristics. Hence, a concise review of several commercial PE-HD grades directed to such applications is also presented in the following sections of this paper together with an evaluation of the applicability of the recyclate for transport packaging as a predefined application.

### 2.2. Material Processing

**Figure 1:**
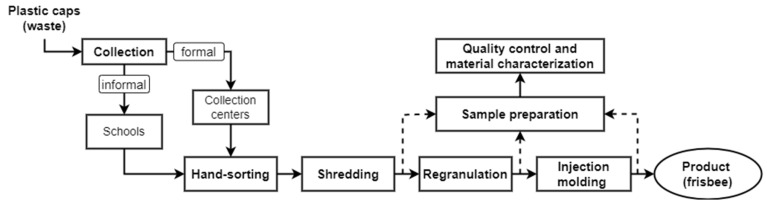
Flow chart of the processing steps of an open-loop recycling process—from beverage bottle caps to a flying disc (i.e., frisbee).

Figure 1 schematically illustrates the recycling process steps of the defined input waste stream, in this case the PE-HD bottle caps. The main step in mechanical recycling is regranulation. To perform this step on the available recycling extruder at the LIT Factory, a minimum amount of approximately 400 kg of the defined input waste stream is required. In order to meet this requirement within the timeline of the project, two collection methods had to be employed. The first was informal collection, where end-users are responsible for separating and gathering the targeted materials. The second was formal collection, which involved the use of standard collection techniques. As a result, the objectives of the study were broadened to allow for a comparison between the two collection strategies. In the informal approach, materials were collected over a two-month period by students and pupils in the district of Upper Austria. For this purpose, collection boxes were distributed to 103 classes at 19 different schools and facilities of other educational institutions (e.g., university campuses) together with the instruction to only dispose of beverage bottle caps made of PE-HD. In the formal approach, waste materials were collected and pre-sorted to remove metal contaminants by the waste collection centers in Upper Austria. Afterwards, the designated “PE-HD bottle caps” fraction was provided by the Upper Austrian head agency for waste treatment LAVU “O.Ö. Landes-Abfallverwertungsunternehmen GmbH” (Wels, Austria).

**Figure 2:**
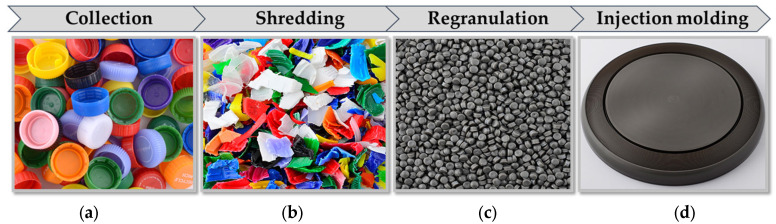
Images of the materials after the different processing steps: (**a**) sorted PE-HD bottle caps, (**b**) shredded PE-HD flakes, (**c**) rPE-HD recyclates, and (**d**) product (frisbee).

In total, approximately 486 kg of seemingly PE-HD beverage bottle caps was gathered. More than half of the amount came from the informal collection (schools). The remaining amount, which was approximately 235 kg, was delivered via formal collection. Before entering the recycling process, both material fractions were hand-sorted based on a set of criteria to sort out any discernible contaminants, thus enhancing the material’s purity. Materials were rejected if they were non-polymeric (e.g., metals); labeled caps; multi-component caps; and caps that were clearly from non-beverage applications or not made of PE-HD. After the hand-sorting step, roughly 10% of the input was discarded, resulting in a sorted stream of around 435 kg that was used in the following recycling steps. The sorted fraction was then shredded into flakes by an industrial single-shaft Micromat 1500 shredder (Lindner-Recyclingtech GmbH, Spittal an der Drau, Austria). Subsequently, the flakes were converted into PE-HD recyclates (rPE-HD) on an INTAREMA 1108 TVEplus recycling extrusion system equipped with filtration and degassing units (EREMA Group GmbH, Ansfelden, Austria). In a typical recycling process, a washing step is commonly employed prior to the extrusion process. However, in this study, the washing step was excluded due to constraints in washing large quantities of input materials within a reasonable timeframe. Moreover, the visual inspection of the input stream revealed that the materials only had minor surface contaminations originating from beverage residues. These residues generally produce minimal volatiles during processing, which have a negligible impact on the mechanical properties of the recyclates. Therefore, the influence of these minor contaminations was neglected in this study. Since no color-based sorting was carried out on the input waste stream, the regranulation process yielded recyclates with a dark grey color (see Figure 2c). Finally, the recyclates were converted into the products (i.e., the frisbees) using an Engel Duo 350 injection-molding machine (Engel Austria GmbH, Schwertberg, Austria). Images of the various material states are illustrated in Figure 2 along with a summary of their information, which is tabulated in Table 1.

### 2.3. Material Characterization Methods

#### 2.3.1. Sample Preparation

Sample preparation refers to the processes carried out on a material to make it suitable for analysis, such as size reduction, extraction, and homogenization. The specific steps involved in sample preparation depend on the nature of the material and the used analytical technique [25,26]. Material characteristics are highly affected by intrinsic and non-intrinsic factors including its state, location, degree of homogeneity, size, etc. [27,28]. Hence, a reliable sample preparation procedure is a prerequisite to produce a representative and homogeneous sample that can be easily analyzed and can adequately reflect the composition of the original material fraction. Therefore, to improve the homogeneity of the input materials after shredding, samples of both input fractions underwent an additional size reduction step by a Retsch SM 300 cutting mill (Retsch GmbH, Haan, Germany) with a rotor speed of 1200 rpm and maximum output particle size of 4 mm. The milled materials were then used, along with the recyclates, for measurements where no further processing is necessary, such as melt mass-flow rate (MFR).

For the other measurements that require the production of test specimens, for instance tensile and impact specimens, the processing technology was determined in accordance with the standard ISO 17855-2 [29]. The standard dictates that test specimens of molding and extrusion PE grades with an MFR of ≥1 g/10 min and ≤1 g/10 min should be produced by injection molding and compression molding, respectively. The MFR measurements of the input materials, which are discussed in the following chapter, revealed that the values lie within the first range (i.e., ≥1 g/10 min). Therefore, multipurpose specimens (MPSs) of type A1 of both milled input materials and the recyclates were injection-molded using a Victory 60 injection-molding machine with a 25 mm cylinder (Engel Austria GmbH, Schwertberg, Austria) in compliance with ISO 3167 and ISO 294-2 [30,31]. Since the materials’ MFR was found to be higher than 1 g/10 min, the processing temperature (i.e., melt temperature) was set at 210 °C as recommended in ISO 17855 [29]. Similarly, type 1 specimens for Charpy impact tests, as described in ISO 179-1 [32], of the input materials and the recyclates were also injection-molded in accordance with the same standards. Prior to testing, all injection-molded specimens were preconditioned for 3 up to 5 days at room temperature (i.e., 23 °C) and 50% relative humidity as recommended in ISO 291 [29,33]. Additionally, to ensure a high level of homogeneity, other measurements were also performed on the cross section of MPSs.

#### 2.3.2. Material Processability

The following test methods were carried out to better understand the material processability. Although they are not intrinsic material characteristics, flake size distribution (FSD) and apparent density (AD) measurements were performed since they are characteristics of material uniformity. Thereby, they can provide information that indicates the material’s ability to be fed into an extruder [34,35]. Moreover, MFR measurements were performed to determine the compatible processing parameters of the input material stream at the various processing steps. The values were also used as an indicator of induced material degradation due to processing.

**Flake size distribution:** The FSD was determined according to the test method B in the standard guide ASTM D1921 [34], which is directed to materials with irregular particle size distribution. The analysis was carried out on the flakes after shredding by an automatic vibratory sieve shaker (model AS 200 control B) manufactured by RETSCH GmbH (Haan, Germany) with defined particle size ranges. Due to the asymmetric geometry of plastic flakes, a particle is considered from a size span once two of its dimensions fall into the respective range. The screen sizes were chosen to cover the ranges of interest as 2.0, 4.0, 8.0, and 11.2 mm. The sieve screens were stacked with the coarsest sieve screen on top to the finest on the bottom pan. After mixing properly, a sample of approximately 1000 g of shredded flakes from each waste source (i.e., schools and collection centers) was collected for the analysis. Each sample was divided into portions of 100 ± 5 g to avoid clogging the screens. Finally, the retained material fractions on each sieve screen were weighed to the nearest 0.01 g by a digital laboratory scale. These weights were then used to calculate the percentage of each size span.

**Apparent density:** The ADs of the shredded flakes of the two differently collected waste streams (formal and informal) as well as that of the recyclates were determined based on the test method C in the standard guide ASTM D1895 [36]. This method is specifically designed for molding materials that are supplied in irregular forms, such as flakes, chips, fiber cuts, etc. However, to maintain a good level of comparability, the same method was performed on the recyclates after regranulation. The measurement tool consists of a cylinder with a volume of 1000 cm^3^ and a hollow cylinder closed at one end (plunger) with a slightly smaller outer diameter than the inner diameter of the first cylinder. The tool was self-constructed by 3D printing PLA material with fused deposition modeling technology. To measure the AD, the measuring cylinder is placed on a flat surface and then 60.0 ± 0.2 g of the material is poured into it. Afterwards, the plunger is loosely placed onto the material to measure the height of the volume that the material occupies in the cylinder. The measurement is performed without and with a weight load of 2300 ± 20 g (including the weight of the plunger). The additional weight can be inserted into the plunger. After measuring the heights (h_1_) and (h_2_), without and with the weight load, respectively, the AD is calculated as shown in Equation (1):

(1) $$V=h\;\cdot \;A\;\mathrm{AD}=\frac{W}{V}\;\mathrm{or}\;\mathrm{AD}=\frac{W}{h\;\cdot \;A}$$

where V is the volume occupied in the cylinder, h is the height of the material in the cylinder, A is the inner cross-section area of the cylinder, and W is the weight of the poured material.

This procedure was repeated on five different samples per material, and then the mean value and the standard deviation of the AD were calculated. Additionally, the bulk factor of both input streams was calculated with respect to the AD of the resulting recyclate as follows:

(2) $$\mathrm{Bulk}\;\mathrm{factor}=\frac{{\mathrm{AD}}_{\mathrm{output}}}{{\mathrm{AD}}_{\mathrm{input}}}$$

**Melt mass-flow rate:** To assess the materials’ processability, melt mass-flow rate (MFR) measurements were performed at each processing step by an Aflow extrusion plastometer instrument manufactured by ZwickRoell Group (Ulm, Germany). To ensure a good representation of the material property, three samples of approximately 4 g of each material (i.e., milled flakes, recyclates, and milled products) were tested. The measurements were performed under predefined test conditions according to the displacement measurement method (method B) in the standard ISO 1133-1 [37] The test temperature was set to 190 °C. After filling the cylinder, the material was automatically compressed by a piston under a load of 2.16 kg to flow through a die with a nominal height of 8 mm and a diameter of 2.095 mm after a preheating time of 300 s. Afterwards, based on the vertical displacement of the piston, six extrudates were cut and weighed for the calculation of the MFR values.

#### 2.3.3. Thermal Analysis

**Ash content:** The ash content (AC) test is typically used for the determination of inorganic residues in plastic materials. Inorganic residues can influence the mechanical properties of plastic products depending on their size, content, and distribution in the bulk. Hence, this method is widely used in the plastics recycling industry since the content of inorganic residues of the resulting recyclates varies depending on the input stream [38]. In the present case study, the AC was determined after each processing step starting with the shredded flakes and ending with the product. The analysis was performed according to method A for rapid ashing as described in the standard ISO 3451-1 [39]. Ashing was carried out in quartz fiber crucibles with a Phoenix microwave muffle furnace manufactured by CEM (North Carolina, USA), and three different samples of each material were tested to calculate the mean and standard deviation values. A test portion of approximately 3 ± 0.2 g of each material was introduced into the crucibles. Samples were directly calcinated in the microwave furnace at 750 °C for 15 min. Subsequently, the crucibles were weighed, and Equation (3) was used to calculate the ACs:

(3) $$A\%=\frac{m_{1}}{m_{0}}\;\times \;100\;$$

where A% is the resulting AC, m_0_ is the initial mass of the test sample, and m_1_ is the measured mass of the obtained ash.

**Differential scanning calorimetry:** A differential scanning calorimeter (DSC) DSC 8500 (PerkinElmer Inc., Waltham, MA, USA) was used for the thermal analysis of the materials at the different stages of the recycling process. Measurements were performed on samples taken from the MPS of the flakes and recyclates to ensure a high level of homogeneity, whilst the samples of the product were taken directly from the frisbees. Hence, samples of approximately 8 ± 1 mg were cut from the cross section of the injection-molded MPS and from the frisbees. Three individual samples of each material state were then encapsulated in aluminum pans. A dynamic temperature scan program was defined for the measurements, which consisted of an initial heating step, subsequent cooling, and second heating step. All three steps were performed over a temperature range from 0 °C to 300 °C and with a constant heating/cooling rate of 10 K/min. To maintain an inert atmosphere and avoid oxidation, the instrument was purged with nitrogen during the measurement with a constant flow rate of 20 mL/min. DSC measurements were performed to identify the melting peaks of the bottle cap materials in the second heating scan, as well as to show possible cross-contaminations of other polymers. Moreover, the melting enthalpy of the present polymers was determined through the integral of the area of the visible melting peaks on the thermograms over the temperature range from 60 °C to 137 °C and from 138 °C to 168 °C for the PE and PP fractions, respectively. To avoid the induction of a systematic error due to sample variation, the heat flux was normalized by the sample mass. The measurements and data evaluation were conducted in accordance with the standards ISO 11357-1 and ISO 11357-3 [40,41].

**Oxidation induction temperature:** A differential scanning calorimeter DSC 4000 (PerkinElmer Inc., Waltham, MA, USA) was used to measure the oxidation induction temperature (OIT). This measurement is an indicator of the thermal stability of materials and their resistance to oxidative decomposition. Hence, OIT measurements took place only after the processing steps, to evaluate the induced degradation from each step. Similarly, samples were cut from the granulates, from the shoulders of the injection-molded MPS, and from the frisbees. Three samples of approximately 8 ± 1 mg, taken from three individual specimens of each material state, were encapsulated in perforated aluminum pans. A dynamic temperature scan program was defined for the measurement. The program starts with an initial isotherm of 1 min at 30 °C, and then the temperature increases with a constant heating rate of 10 K/min to 260 °C. Synthetic air was used as purge gas with a constant flow rate of 20 mL/min. The heat flux of the measurements was normalized by the individual sample masses; thus, the normalized thermograms were used for the evaluation. OIT was determined as the intercept point between the two tangents of the onset of exothermic oxidation on the thermogram. The measurements and the data evaluation of the dynamic OIT were conducted according to ISO 11357-1 and ISO 11357-6 [40,42].

#### 2.3.4. Mechanical Testing

**Tensile tests**: Mechanical properties are essential for numerous applications. Therefore, tracking the changes in the tensile properties of the materials throughout the whole recycling process was of interest to this project. As previously described in Section 2.3.1, MPSs of both input materials as well as the recyclates were produced via injection molding, whereas type 5 specimens, which are depicted in ISO 527-3 [43], were punched out of the flat surface of the frisbees to determine their tensile properties. Considering the radial flow of the polymer melt due to the mold geometry, specimens were punched in the transverse direction to ensure an adequate level of consistency. As mentioned above, specimens were stored for 3–5 days before the tests at 23 °C and 50% relative humidity according to ISO 291 [29,33]. Moreover, ten specimens of each material were examined to achieve a good representation of the material properties. All tests were performed at 23 °C using a universal testing machine Zwick/Roell AllroundLine Z005 equipped with a multiXtens strain measurement system produced by ZwickRoell Group (Ulm, Germany). The test parameters were set for MPSs according to ISO 527-1 and ISO 527-2 [40,41,44,45] with a transverse speed of 1 mm/min for the determination of the tensile modulus until a strain of 0.25% is reached. Afterwards, the speed is increased automatically to 50 mm/min and kept constant until failure. For the type 5 punched specimens, the test parameters were defined in accordance with ISO 527-1 and ISO 527-3 [39,40,43,44]. However, to maintain comparable results with the other material states, the test speed was calculated based on the strain rate of the selected parameters for MPSs and then rounded to the nearest recommended values in ISO 527-1. Thereby, the transverse speed was set to 0.5 mm/min for the determination of the tensile modulus and then increased to 20 mm/min until failure for the calculation of the other tensile properties.

**Charpy impact tests:** To have a broader overview of the mechanical properties of the materials, non-instrumented Charpy notched impact tests were performed on both input and output materials using an HIT25P pendulum impact tester from ZwickRoell Group (Ulm, Germany). After the production of type 1 impact specimens, as described in Section 2.3.1, a type A V-shaped notch (45° ± 1° notch angle, 0.25 ± 0.05 mm notch tip radius), as depicted in ISO 179-1 [32], was cut into the specimens using an RM2265 automatic rotary microtome (Leica Biosystems Nussloch GmbH, Nussloch, Germany). To have a better understanding of the material behavior, specimens were conditioned for 24 h before testing at 23 °C and 50% relative humidity [33] as well as at −20 °C. A set of ten conditioned notched specimens of each material state was tested, with tests being conducted edgewise and in accordance with ISO 179-1 [32]. Then, the mean values and the standard deviations of each material were calculated.

Table 2 provides an overview of the various test methods that were used in this study with an indication of the material states on which each method was performed.

### 2.4. Review of Selected Commercial Packaging Grades

Due to the extensive use of plastics in a broad range of applications, plastics are available with a high diversity of technical properties. Polymers are versatile materials whose characteristics are not solely influenced by their chemical composition but also by their molecular structure. Hence, varying the molecular structure of a polymer during the polymerization process results in new subtypes, often referred to as grades [46,47]. The selection of a material grade with the right processing and mechanical properties for a certain product primarily depends on the information provided on the technical product datasheets (TDSs). Hence, understanding the technical performance of a material is of profound importance to effectively use it in a specific application. PE-HD grades that are specifically intended for the production of plastic beverage bottle caps are widely available in the product portfolios of most PO producers [15]. To gain a fundamental understanding of the property profile of these grades, an incomprehensive yet illustrative review of TDSs of selected PE-HD grades was conducted. Since this study is based on an open-loop recycling process due to the strict food-contact regulations, the review was expanded to include another packaging application, for which such a waste stream can potentially be utilized as a full or partial substitution of virgin grades. Materials that are used for transport packaging (TP) applications, such as boxes and crates, were chosen as the target benchmark material. These applications have specific material requirements that can vary depending on their intended use. They should, in general, be made of durable and impact-resistant materials to withstand the loads of stacking and handling during transportation and storage. Additionally, they should be made of lightweight plastics, such as PE-HD or PP [48,49]. The review surveyed TDSs of 29 distinct commercial PE-HD grades provided by three major material suppliers—including Borealis Polyolefins (Vienna, Austria), SABIC (Riyadh, Saudi Arabia), and LyondellBasell (Rotterdam, the Netherlands) [50,51,52,53]. The grades encompassed 23 and 10 application-specific types, which are suitable for caps and closures (CC) and TP products, respectively, with four overlapping types that can be used for both applications. The results of the survey are shown in a later section in this article.

### 2.5. Material Assessment Using the Substitution Potential Concept

To determine if the investigated waste stream is suitable for the proposed application, an application-specific comparison was conducted between the property profile of the beverage bottle cap recyclate (i.e., rPE-HD) to those of selected commercial virgin grades that are used for TP products. This comparison was based on the data compiled from the reviewed TDS documents and the substitution potential (SP) concept. The SP is the ability of one material to replace another in a particular application or use case based on technical, environmental, and economic factors [47,54,55]. However, in case of plastics recycling, a full replacement is often not feasible, mostly due to the insufficient quality of the recycled plastics [56]. Hence, several research studies have emphasized the significance of taking the technical quality of the recycled plastics into consideration at early stages of the recycling process [10,47,55,57,58]. For example, Roithner and Rechberger [58] highlighted in their paper the significance of incorporating the quality dimension in the overall understanding of plastics recycling. They argued that the conventional definitions of recycling rates do not reflect the complete picture of the environmental benefits of plastics recycling as they are solely quantitative without counting the material quality as a factor. Hence, they proposed a new approach to calculate the recycling rates that combines both quantitative and qualitative recycling aspects. Another study from Eriksen et al. [59] examined the ability of various recovery systems to close material loops based on the circularity potential and the contamination level of resulting waste fractions. While the techniques described in these studies can significantly enrich the information on recycling performance, they fall short in establishing a basis for a strategy to effectively estimate the SP of recycled plastics. This is because their methods only consider the material’s purity as a quality parameter and fail to consider other technical or functional material characteristics. In contrast, other studies have presented elaborate methods for the SP of recycled materials. For instance, Vadenbo et al. [55] proposed a systematic reporting framework to assess the potential resource recovery of a waste stream and to systematically estimate its SP. Moreover, they distinguished between the terms substitutability and substitution potential, as they defined substitutability as the degree of functional equivalence between alternative resources for a specific application, whereas, in their concept, the SP (γ) is described as a function of four key components (shown in Equation (4)), including (1) the physical resource potential ($U^{\mathrm{rec}}$), which is the proportion of the desired material in a waste stream, (2) the resource recovery or recycling efficiency ($\eta^{\mathrm{rec}}$), which is the expected yield of the employed waste management system, (3) the substitutability ($\alpha^{\mathrm{rec}:\mathrm{vir}}$), which represents the functional performance of a recycled material compared to that of a virgin grade for a specific end-use or product, and (4) the market response ($\pi^{\mathrm{vir}}$), which is the anticipated displacement level of virgin materials by their secondary or recycled counterparts.

(4) $${\gamma =U}^{\mathrm{rec}}{\cdot \eta }^{\mathrm{rec}}{\cdot \alpha }^{\mathrm{rec}:\mathrm{vir}}{\cdot \pi }^{\mathrm{vir}}$$

Vadenbo et al. [55] suggested a method to calculate the substitutability factor ${\;\alpha }^{\mathrm{rec}:\mathrm{vir}}$ as the ratio of an application-specific functionality of a recycled resource (e.g., recycled plastic) over that of each potentially displaced resource (e.g., virgin grades). Demets et al. [47] raised concerns about this method, suggesting that it could oversimplify or overlook some functional aspects of an application, since it only relies on one main property that typically characterizes a key function of that specific application. Therefore, they proposed a new approach to calculate $\alpha^{\mathrm{rec}:\mathrm{vir}}$ that takes into account the material processability and adjusts the appreciation of the different mechanical properties according to the nature of the intended application. Their approach relies on two quality components to determine the technical substitutability of a material. One is process-related (${\mathrm{RQ}}^{\mathrm{proc}}$), and the other is mechanical-property-related (${\mathrm{RQ}}^{\mathrm{mech}}$). This approach is used in this paper to calculate the SP of the bottle cap recyclate rPE-HD as a measure to determine if the material is suitable for the predefined application, namely TP products.

**Figure 3:**
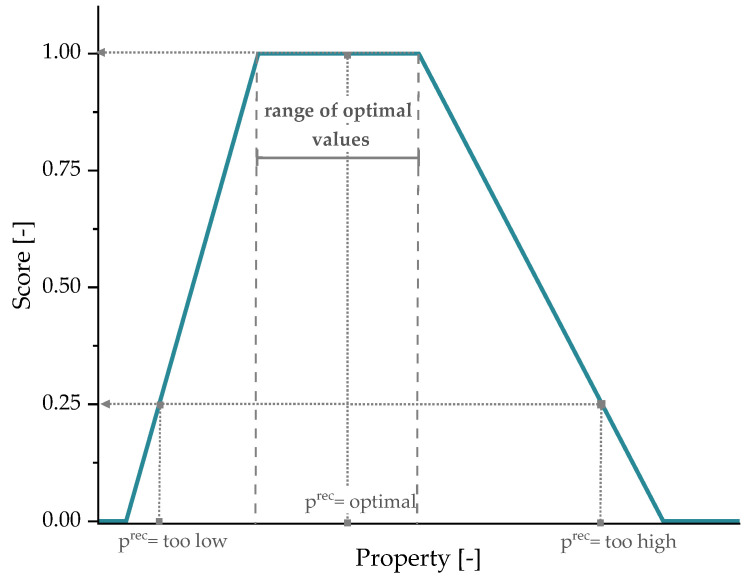
An exemplary trapezoidal function which is used for the performance scoring of the various material properties including the optimal range derived from the TDS of PE-HD virgin grades and based on the method presented by Demets et al. [47] (own illustration).

According to Demets et al. [47], to handle the application-dependent functionality, it is necessary to establish scoring functions (f) for the relevant properties of a specific application. Such scoring functions can be quantified according to the material’s requirements provided by the converters or based on the typical values of the essential properties of virgin materials that are used for that specific application. Thereafter, a score ranging from 0 to 1 will be assigned to each recyclate’s property depending on how much it deviates from the defined range, with 1 being within the optimal range and 0 being utterly outside of it. An example of such functions is depicted in Figure 3, which shows a trapezoidal function with upper and lower constraints, thereby a too high or too low value leads to a lower score. This applies to properties that cannot tolerate deviation from the specified range in both directions, such as tensile modulus and MFR [47,60].

For instance, in thermoplastic extrusion, a high MFR could result in catastrophic outcomes, while having high MFR is favorable in injection molding to ensure a proper mold filling. Hence, MFR serves as the first parameter to assess if the intended technology is capable of processing the material. Other material properties such as strength, strain, and toughness only have a lower threshold to be fulfilled [60]. Therefore, a scoring function with only a lower boundary can be sufficient. Accordingly, the material processability is denoted ${\mathrm{RQ}}^{\mathrm{proc}}$ and defined by a scoring function f (see Equation (5)). The MFR $F^{\mathrm{vir}}$ defines the processing window for the desired application, within which a material can attain a score between 0 and 1, and $F^{\mathrm{rec}}$ recyclate value should fall within this range depending on its MFR.

(5) $${\mathrm{RQ}}^{\mathrm{proc}}{=f\;(F}^{\mathrm{vir}}{,\;F}^{\mathrm{rec}})$$

On the other hand, the functional quality of the material, described by the mechanical-property-related parameter ${\mathrm{RQ}}^{\mathrm{mech}}$, is calculated as the sum of the weighted scores of the recyclate’s mechanical properties that are derived from the scoring functions of the desired properties of virgin grades $P_{i}^{\mathrm{vir}}$ (Equation (6)). To illustrate, if we choose to calculate ${\;\mathrm{RQ}}^{\mathrm{mech}}\;$ based on the four essential mechanical properties $P_{i}^{\mathrm{rec}}$, including tensile modulus (E), stress at yield ($\sigma_{y}$), notched impact strength ($a_{\mathrm{cN}}$), and strain at break ($\varepsilon_{b}$), Equation (6) can be rewritten as demonstrated in Equation (7).

(6) $${\mathrm{RQ}}^{\mathrm{mech}}=\sum_{i=1}^{n}w_{i}{\cdot \;f\;(P}_{i}^{\mathrm{vir}}{,\;P}_{i}^{\mathrm{rec}})$$

$$\mathrm{where}:\;\sum_{i=1}^{n}w_{i}=1$$

(7) $${\mathrm{RQ}}^{\mathrm{mech}}{=w}_{E\;}\cdot \;f\;\left(E^{\mathrm{vir}}{,\;E}^{\mathrm{rec}}\right){+w}_{\sigma }\;\cdot \;f\;\left(\sigma_{y}^{\mathrm{vir}}{,\;\sigma }_{y}^{\mathrm{rec}}\right){+w}_{a}\;\cdot \;f\;\left(a_{\mathrm{cN}}^{\mathrm{vir}}{,\;a}_{\mathrm{cN}}^{\mathrm{rec}}\right){+w}_{\varepsilon \;}\cdot \;f\;\left(\varepsilon_{b}^{\mathrm{vir}}{,\;\varepsilon }_{b}^{\mathrm{rec}}\right)$$

Nevertheless, it is necessary to adjust the scoring function f of each mechanical property $P_{i}$ and the corresponding weights $w_{i}$ based on the specific application. Hence, the scoring function must indicate the extent to which an increase or decrease in a property is unfavorable in the defined application, while the weights should prioritize the properties according to the functionality of that application. Finally, the substitutability factor is determined as the minimum of the two resulting RQ parameters, as shown in Equation (8). This ensures that a recyclate can be considered for an application, only in case of compliance with the application requirements in terms of both processability and functionality.

(8) $$\alpha^{\mathrm{rec}:\mathrm{vir}}{=\min\;[\mathrm{RQ}}^{\mathrm{proc}}{,\;\mathrm{RQ}}^{\mathrm{mech}}]$$

## 3. Results

### 3.1. Material Evaluation

#### 3.1.1. Material Processability

**Figure 4:**
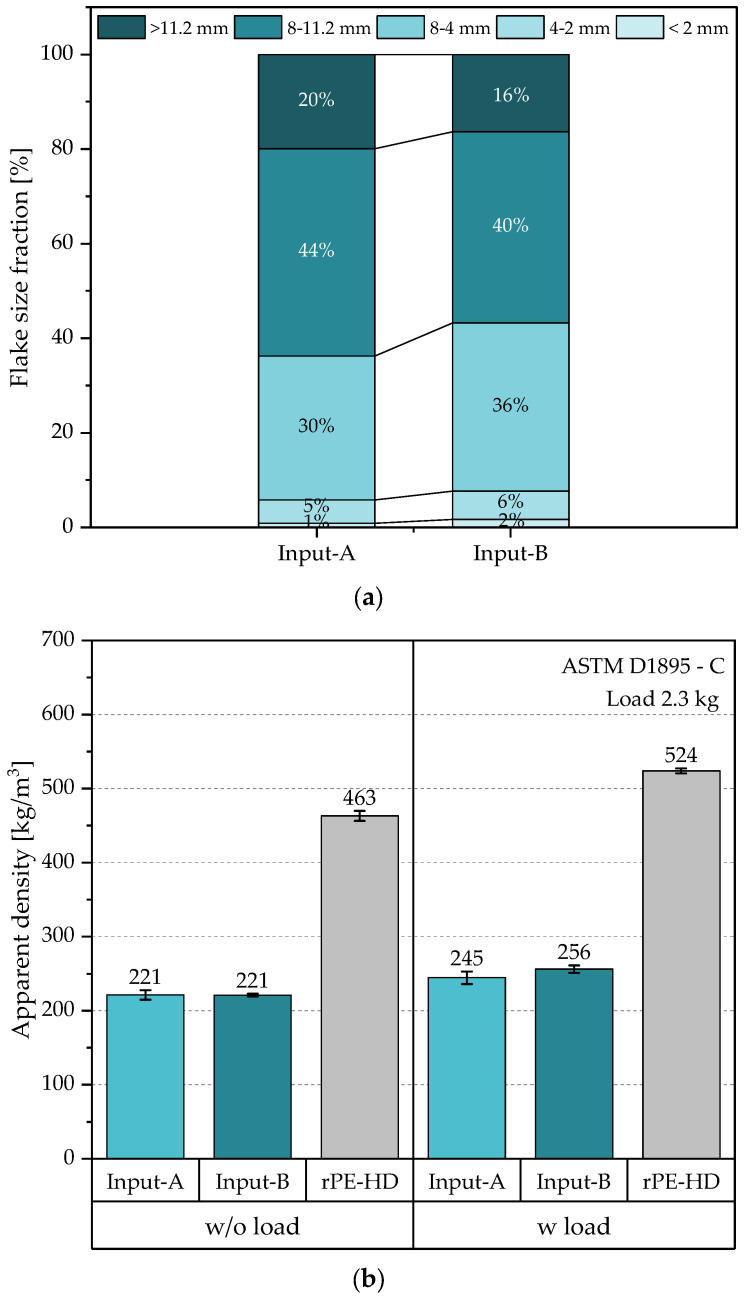
Flake size distribution (FSD) of the shredded input fractions (**a**), along with their apparent density (AD) in comparison to that of the resulting rPE-HD pellets (**b**).

To evaluate the processability of the various processing steps, the FSD, the AD, and the MFR of the different material states were measured. Unlike the MFR, the AD and the FSD are non-intrinsic material characteristics [27,36]. They both, however, can indicate the material uniformity, which makes them useful for assessing the handling and processability of a material, particularly how well it can be fed into an extruder [34,35]. While the AD defines the volume filled by a material in a confined space, the FSD refers to the particle size distribution of the material. Typically, the shredding step yields flakes of varying sizes and shapes leading to a dimensional heterogeneity in the resulting flakes [61]. This heterogeneity affects the material’s AD as it is influenced by the geometry of the particles (i.e., the flakes). Moreover, it may also cause fluctuations in the extrusion step due to possible segregation based on size or density, which results in material bridging in the feeding unit and thus a shortage of material feed into the extruder [27,35]. Figure 4 graphically illustrates the FSD and the AD of both input streams and the resulting recyclate.

The retained size fractions on the sieve screens of the vibratory shaker were analyzed to obtain the FSD for both input streams. Figure 4a shows that both samples of the shredded input streams comprised flakes from all defined size spans and had comparable FSDs. Most of the flakes in both samples were larger than 2 mm, and only a small proportion of less than 2% was considered as a fine fraction (i.e., flake size <2 mm). A little over 5% of each sample fell into the size range of 2–4 mm. This was followed by the size range from 4 to 8 mm accounting for 30% of Input-A and 36% of Input-B. The majority of flakes in both samples had sizes between 8 and 11.2 mm, accounting for 44% and 40% of Input-A and Input-B, respectively. The remaining fractions of both input streams had a flake size larger than 11.2 mm and accounted for 20% and 16% of Input-A and Input-B, respectively.

The usual AD values of PO pellets (i.e., PE and PP) range from 500 to 580 kg/m^3^. In case of other material geometries, this value drops to as low as 130 kg/m^3^ for PE films and to around 350 kg/m^3^ for rigid flakes [35,62]. These values may, however, still vary depending on some factors, such as the shape and size of the flakes and the actual material density [27,62]. The average AD values of both input streams along with those of the resulting recyclate are presented in Figure 4b. It is evident that both input streams had the same AD value when no weight load was applied. This value increased to double as high value in the resulting recyclate, precisely with a bulk factor of approximately 2.1. Obviously, when the weight load was applied, the input streams were compressed, resulting in an increase of about 11% and 16% in the AD values of Input-A and Input-B, respectively. The slightly higher increase in AD of Input-B can be attributed to the relatively smaller flakes of this stream resulting from shredding, as around 44% of this material had a flake size below 8 mm compared to 36% in the other material. Therefore, the possibility of a better filling of voids when a weight is applied is higher in this material. Similarly, the AD of the recyclate also increased by 13% due to the compression by the weight load corresponding to a bulk factor of 2.1 on average. In general, both material states showed AD values slightly lower than those reported in the literature. However, no issues emerged during processing that could be attributed to lower AD values.

**Figure 5:**
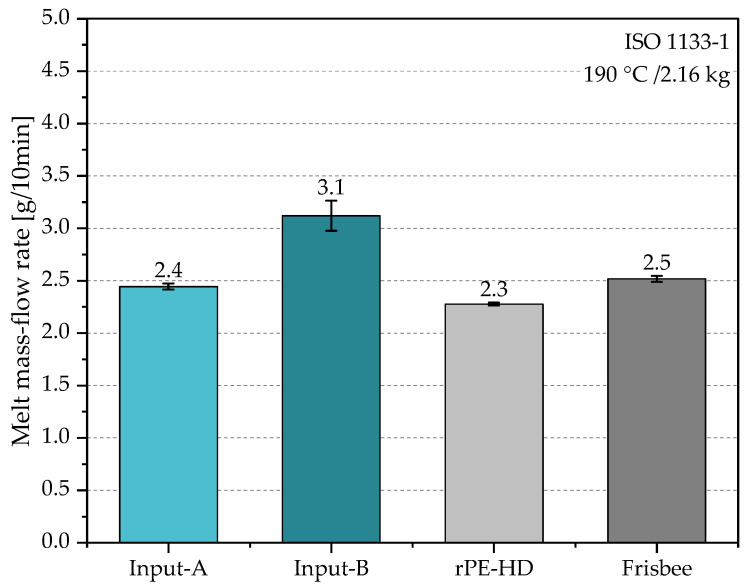
Measured melt mass-flow rate (MFR) values of the investigated materials.

As mentioned before, to assess the materials’ processability and to monitor any potential alterations in the material due to degradation, MFR measurements were carried out at different stages of the recycling process. In contrast to complex viscosity measurements, MFR offers single-point data that adequately indicate the processability of a material by a certain technology. Thus, it is crucial in the production of thermoplastics. The experimentally determined MFR values of the investigated materials are depicted in Figure 5. The MFR of Input-A is 23% lower than that of Input-B. Given that both material streams were unwashed, this might be due to a higher amount of surface contamination in the formally collected materials (i.e., Input-B). Another explanation for the increased MFR could be due to cross-contamination by other polymers of higher MFR (e.g., PP). However, these differences were not reflected in the MFR of the resulting recyclate, which was even slightly lower than that of Input-A. This can be due to the filtration unit that may have filtered out some contaminants that initially raised the MFR of the input materials. Moreover, it can also be linked to the usual degradation mechanisms of PE-HD which cause the macromolecular chains of the polymer to branch and eventually crosslink, thereby elevating its viscosity [63]. To fully trace the material’s property profile, MFR measurements were also performed on the products (i.e., frisbees). However, to make the material suitable for the measurement, it was necessary to perform a size reduction step on them. Randomly selected frisbees were shredded and milled in a similar fashion as the input streams to produce particles with the appropriate size (see Section 2.3.1). The MFR value of the milled frisbee seemed to be 4% and 8% higher than that of Input-A and rPE-HD, respectively, and 20% lower than that of Input-B. Presumably, this variation in the MFR values can be explained by the initial level of contamination of the input materials and the uneven distribution of contaminants during the regranulation process.

#### 3.1.2. Thermal Properties and Stability

**Figure 6:**
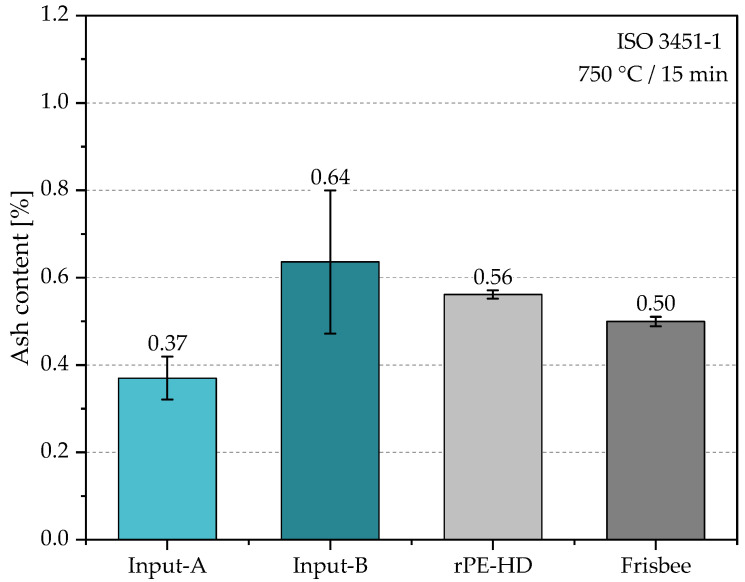
Graphical illustration of the ash content (AC) of the various material states.

Materials were subjected to thermal analyses for two primary purposes. Firstly, to qualitatively assess the contamination level throughout the various processing steps and to identify the residual contaminants present in the material. Secondly, to provide an indication of degradation induced by processing through the assessment of the inherent thermal stability resulting from the effectiveness of the residual stabilizer in the material as well as from the degradation state of the material itself. Figure 6 shows the AC of the investigated materials, which were generally characterized by relatively low levels of inorganic contaminants, with all values being below 1%. The AC of Input-B (0.64%) was a little over 40% higher than that of Input-A (0.36%), which can possibly be due to a higher surface contamination. This suggests that the informal collection method may have yielded a cleaner input stream (i.e., Input-A). Obviously, the slightly higher AC of Input-B corresponded to an increase in the AC of the resulting recyclate and subsequently the product, which accounted for 0.56% and 0.50% of their total sample masses, respectively. There is a slight standard deviation between the values of the recyclate and the frisbees, which could be due to an uneven distribution of contaminants in the input stream, especially in Input-B, as can be seen by its high standard deviation. Nevertheless, their values lie within the combined average range of both input streams.

**Figure 7:**
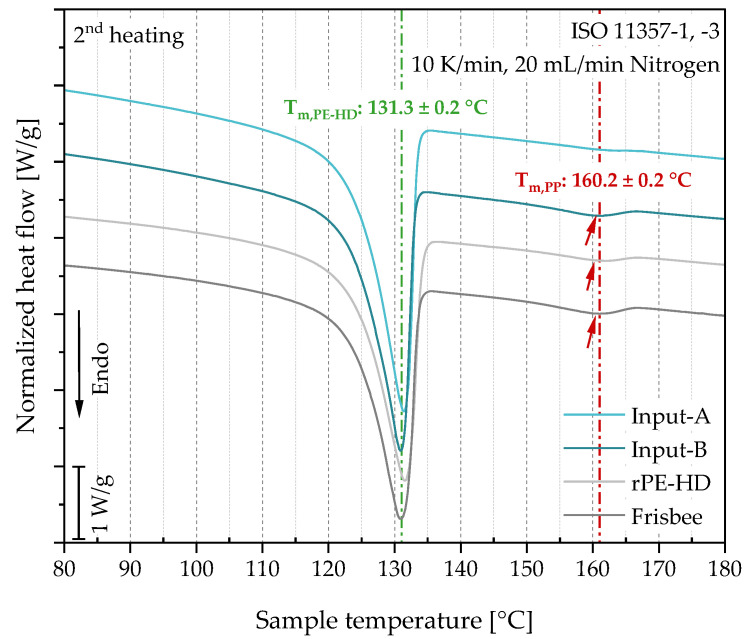
DSC thermograms of the investigated materials showing the melting peak of PE-HD and revealing an obvious PP melting peak in one of the input streams, which suggests a cross-contamination by PP that was passed to the resulting output materials as well.

Furthermore, the DSC measurements revealed a cross-contamination of the input materials by PP. The thermograms along with their numerical interpretation are graphically illustrated and tabulated in Figure 7 and Table 3, respectively. A prominent endothermic peak at around 131 °C can be easily observed on all material curves, which falls within the usual melting temperature range of PE-HD [64,65]. Evidently, this confirms that the targeted polymer, i.e., PE-HD, is the primary component of the input materials and thus the resulting recyclate and frisbees. At higher temperatures, another endothermic peak, yet significantly smaller than that of PE-HD, appears on all curves in the range from 150 °C to 170 °C. This peak corresponds to the melting temperature of PP [66], indicating a cross-contamination by this polymer.

The cross-contamination can be attributed to inadequate separation of the two POs during the collection and sorting steps. While the melting enthalpy of the PE-HD peak in Input-A was approximately 10% higher than that in Input-B, the melting enthalpy of the PP peak in Input-A was considerably lower, measuring only 2.1 J/g (as shown in Table 3) in comparison to 7.7 J/g for Input-B. This suggests that the cross-contamination originated for the better part from Input-B and was subsequently transferred to the output materials.

Furthermore, it is expected that the melting enthalpies of the present polymers in the resulting recyclate fall within the value range of the input streams since the recyclate is a product of both combined. This was observed in both melting enthalpies. However, when examining the values of the frisbees, it can be seen that the melting enthalpy of PE-HD decreased while that of PP increased compared to those of the recyclate. This can be an indicator of changes in the degree of crystallinity of both components present in the material (i.e., PE-HD and PP) since the increase in the melting enthalpy usually corresponds to an increase in the degree of crystallinity and vice versa. These changes can be associated to the degradation of POs caused by mechanical reprocessing. According to Yin et al. [63], mechanical reprocessing inevitably leads to degradation in POs. However, the degradation mechanisms in PE and PP along with their influences on the material properties are different. When reprocessing PE-HD, chain branching and eventually crosslinking take place simultaneously with chain scission, thereby reducing the degree of crystallinity [67,68,69]. In contrast, the degradation in PP due to mechanical reprocessing is dominated by chain scission, which increases the chain mobility and hence the degree of crystallinity. This in turn will be reflected in the other material properties [63,70], which will be investigated in the next sections of this paper. Nevertheless, since the quantity of each PO in the material is unknown and the crystallization mechanism is not solely dependent on degradation, it is difficult to precisely quantify these changes.

**Figure 8:**
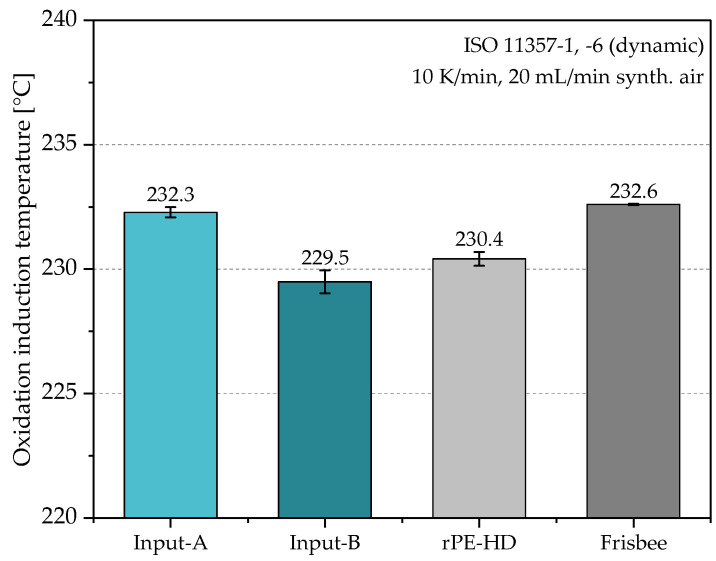
Oxidation induction temperatures (dynamic OIT) of the investigated materials.

The results of the oxidation induction temperature (dynamic OIT) measurements are shown in Figure 8. In this study, since no stabilizing or modifying agents were introduced to the materials during the recycling process, the OIT was used to assess the thermal stability of the tested materials in terms of its resistance to oxidation after mechanical reprocessing. Input-A had an average OIT that was approximately 3 °C higher than Input-B. This difference could be simply attributed to its higher inherent resistance to oxidation due to the presence of residual stabilizers in its materials. Additionally, the higher cross-contamination by PP in Input-B may have also affected its resistance to oxidation, since PP is more prone to oxidation than PE-HD [71]. The OIT values of both the recyclate and frisbee materials were within the same range as the input materials. This suggets that even with the absence of additional processing stabilizers, no significant degradation in terms of the material’s resitance to oxidatation could be observed.

#### 3.1.3. Mechanical Performance

**Figure 9:**
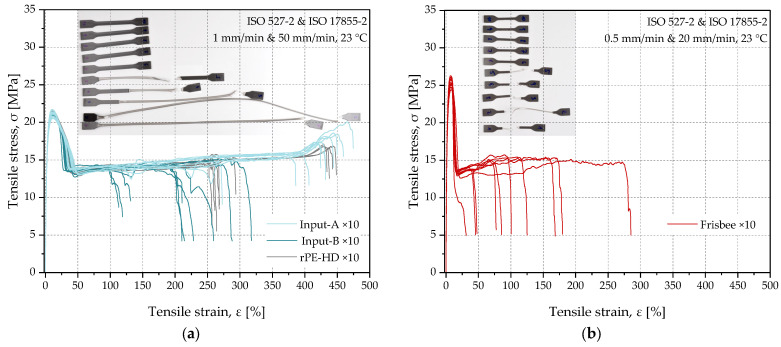
Stress–strain diagrams including exemplary pictures of the specimens prior to and after testing: (**a**) measurements of ten MPSs of each input stream and the recyclates; (**b**) measurements of ten type 5 specimens cut from the flat surface of the frisbees.

The tensile stress–strain diagrams of all materials are depicted in Figure 9. As mentioned in a previous section, while the MPSs of both input streams and the recyclate were used for tensile tests, type 5 specimens were used to measure the tensile properties of the products (i.e., the frisbees). Hence, the stress–strain curves were clustered accordingly. The calculated mean values along with the standard deviations of the essential mechanical properties are listed in Table 4. When comparing the properties of the two input streams, it can be seen that Input-B shows a slightly higher tensile modulus and stress at yield. In contrast, the average strain at the break value of Input-B was by a factor of two lower than that of Input-A. The studies by Gall et al. and Van Belle et al. [72,73] on the structure–property relationships of virgin and recycled PE/PP blends show that the presence of even small fractions of PP in PE-HD leads to a higher tensile modulus and strength and ultimately lower strain at break values. Thereby, the difference in mechanical behavior of the input materials can be explained by the higher cross-contamination of Input-B by PP, which was already demonstrated by the DSC measurements.

After regranulation, both the tensile modulus and yield stress of rPE-HD increased compared to those of the input materials (see Table 4). This processing step was carried out on a recycling extruder equipped with filtration and degassing units that, certainly, enhanced the material’s homogeneity and reduced the amounts of contaminants and volatiles, which could act as crack initiators leading to a premature failure. This could undoubtedly lead to the increase in these two tensile parameters. Furthermore, the presence of the PP fraction resulting from the cross-contamination of the input streams may have also played a role in this increase. As mentioned before in Section 3.1.2, PE-HD and PP have distinct degradation mechanisms during reprocessing [63] that result in changes in the material morphology and molecular structure [68,69,74]. These changes are typically accompanied by changes in the mechanical properties as the tensile modulus and strength of a material increase with increasing degree of crystallinity, whilst the strain at break and the material toughness sink and vice versa [70,72,75]. In case of PO blends, both mechanisms are activated in the respective polymer fractions. Therefore, the increased crystallinity of the PP fraction due to the mechanical reprocessing may have partially contributed to the increased tensile modulus and yield stress of the recyclate.

The stress–strain diagrams of the type 5 specimens punched out of the products are depicted in Figure 9b. On the one hand, the tested specimens showed higher tensile moduli and stress at yield values compared to those of the input streams and the recyclate. This can be explained by the geometry of the specimens and the distinct flow behavior of the polymer melt during the production of the parts (i.e., MPS and frisbees). The radial flow distribution in the frisbee mold introduces a higher shear stress into the polymer melt, leading to a complex orientation of the polymer chains, which affects the mechanical properties of the material [76,77]. However, since the flow modeling of the injection-molded frisbees is not part of this study, this topic will not be further discussed in this paper. On the other hand, the strain at break was considerably lower with a large standard deviation, shown in Table 4. Nevertheless, the standard deviation of the strain at break of all material states was notably high, which compromises the reliability of this material characteristic to be used for the following evaluation steps, namely the application-specific evaluation based on the substitution potential (SP) concept.

Finally, the materials’ toughness, represented by their notched impact strength (NIS), was examined via Charpy notched impact tests. The measurements were performed only on the input materials and the recyclate since the production of comparable specimens from the frisbees was not possible. The NIS values of the materials at two different temperatures are listed in Table 4. At 23 °C, Input-A exhibited the best performance in terms of material toughness exhibiting an NIS of 8.3 kJ/m^2^, which is approximately 9% and 15% higher than those of Input-B and the resulting recyclate rPE-HD, respectively. Presumably, the reduced toughness in Input-B and rPE-HD can also be attributed to the higher cross-contamination by PP, which was previously shown on the DSC thermograms. According to Gall et al. [73], high material toughness of rPE can be achieved only with a very low cross-contamination by PP accompanied with other factors, such as low MFR. Another study by Thoden Van Velzen et al. [78] evaluated the mechanical performance of rPE in relation to the material composition of the feedstock. They reported a clear correlation as a stepwise reduction in toughness with increasing PP content in the recyclate, even at relatively low concentrations. Furthermore, as already established in a previous section, reprocessing PP decreases the material’s toughness, which is associated to the increase in its crystallinity due to reprocessing [63,70]. This explains the further decline in NIS of rPE-HD after the regranulation step. Hence, the notion that the presence of a higher PP fraction in Input-B and rPE-HD led to lowering the material’s toughness can be confirmed. After preconditioning the test specimens at −20 °C, NIS of all three materials decreased, on average, by a factor of two. However, no considerable difference between the three materials was observed. The effect of the lower temperature is therefore more significant than that of any other factor.

### 3.2. Technical Performace of the Recyclate based on Market Information

**Figure 10:**
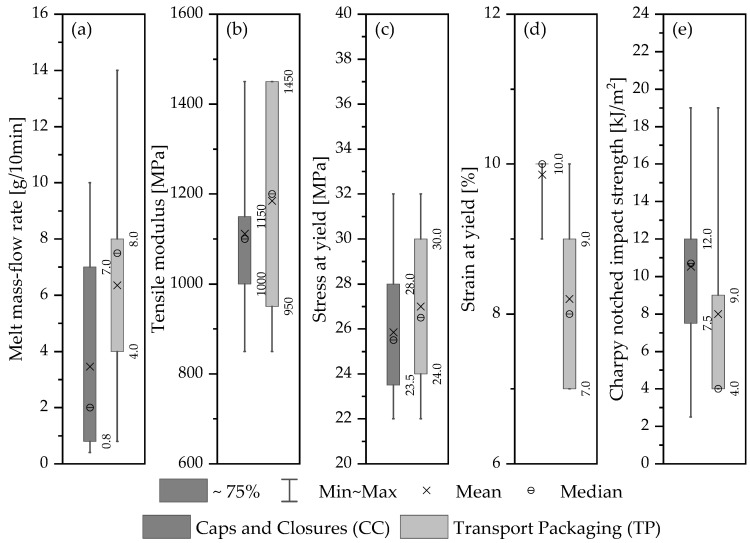
Graphical illustration showing the overlap between the typical ranges of (**a**) MFR, (**b**) tensile modulus, (**c**) yield stress, (**d**) tensile strain at yield, (**e**) Charpy notched impact strength of caps and closures and transport packaging applications based on a review of technical product datasheets (TDSs) of selected commercially available PE-HD grades offered by three major international material suppliers [50,51,52,53] (own illustration).

The technical quality of thermoplastic materials is usually described by their mechanical properties along with their flow behavior in the melt state. The flow behavior of a material, which is usually indicated by the MFR value in the TDS, determines its ability to be processed by a certain processing technique, whereas the primary mechanical properties that are used to describe its overall mechanical performance are typically its strength, stiffness, toughness, and often its ductility [72]. These physical characteristics are normally measured by standardized test methods. The strength, stiffness, and ductility are mostly determined by tensile tests. The material’s ductility is usually approximated by its strain at break and impact strength [47]. However, due to the lack of information on the strain at break in most reviewed TDSs, it was decided for the purpose of this study to substitute it with the strain at yield. Therefore, to quantitatively describe these characteristics of the selected commercial materials, the review focused on: MFR, tensile modulus, stress and strain at yield, and Charpy NIS. Moreover, these measures were also selected because they are presented in a consistent fashion in the reviewed TDSs of the different material grades. Figure 3 illustrates the ranges of properties relevant to caps and closures (CC) and transport packaging (TP) applications. Despite the limited scope of the review, which included only two applications and three material suppliers, the diversity of the materials is substantial, resulting in a broad range of values. However, overlapping ranges between the two applications support the notion of using recycled beverage bottle caps for TP products. In Figure 10a, the MFR values of CC material grades range from 0.4 to 10 g/10 min, with 75% of the material types falling within the range from 0.8 to 7 g/10 min. Whereas TP grades have MFR values ranging from 0.8 to 14 g/10 min, with 75% of the materials’ MFRs lying within the 4 to 8 g/10 min range. The same patterns are observed for the tensile modulus and stress at yield for both applications as the ranges overlap to some extent (Figure 10b,c). On the other hand, in the case of the strain at yield and Charpy NIS, only the complete range indicates an overlap between the values of CC and TP grades, as the range of the former falls within that of the latter. However, when only the 75% range is considered, CC grades lie outside the range of TP grades. This can be attributed to the higher toughness requirements for some CC products, such as bottles for carbonated beverages. Nonetheless, having higher strain at yield and NIS should not hinder the usability of a specific recyclate in a particular product since these are not critical properties and would not negatively affect the final product.

**Figure 11:**
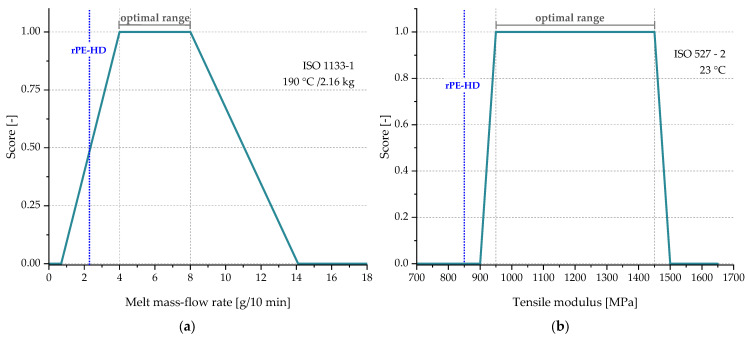

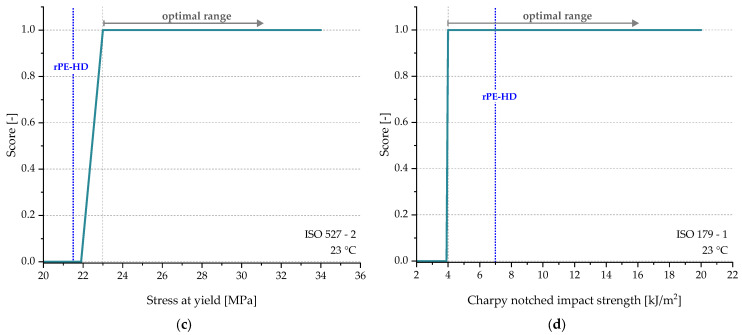
The trapezoidal functions used for scoring (**a**) MFR, (**b**) tensile modulus, (**c**) tensile stress at yield, and (**d**) Charpy notched impact strength (NIS) of rPE-HD, which were generated based on the values from the TDS documents of TP applications. The values lying within the 75% range of the total surveyed data were used to determine the optimal range, and the blue dotted line indicates the average values of rPE-HD.

Based on the reviewed TDSs, scoring functions of the relevant properties were formulated. The minimum and maximum values of the reviewed grades were used to define the upper and lower boundaries, while the targeted optimal range was set as the 75% range, which represents 75% of the available grades for this specific application from the selected supplies. Subsequently, the RQ factors and the α^rec:vir^ were calculated to demonstrate if the recyclate rPE-HD can be used for TP products. The scoring functions are illustrated in Figure 11. As mentioned in Section 2.4, only MFR and the tensile modulus need to be restricted by upper and lower limits; thus, double trapezoidal functions with a defined optimal range were used for these two properties, whereas single functions with only a lower threshold (i.e., minimal required value) were used for the other properties including stress at yield, strain at yield, and NIS. The slope between the boundaries and the optimal range indicates the degree of tolerance for being outside of the optimal range.

Furthermore, the corresponding scores of each property are determined based on the experimental data of the recyclate rPE-HD, which were presented in Section 3.1. The mean values of these properties are represented in Figure 11 by the blue dotted lines. Accordingly, a value of 0.5 can be easily obtained for the factor ${\mathrm{RQ}}^{\mathrm{proc}}$ from the corresponding scoring function and the MFR value of rPE-HD (Figure 11a). However, scoring weights ($w_{i}$) must be applied for the calculation of ${\mathrm{RQ}}^{\mathrm{mech}}$. Demets et al. [47] proposed various weighting schemes tailored for several applications including rigid injection-molded parts (e.g., boxes and crates). One of the recommended schemes assigns the weights to the properties depending on their relative importance for the functionality of the application. Thereby, the weights are distributed in a way that gives the tensile modulus the highest importance with 50% ${(w}_{E}=0.5)$, followed by 30% for NIS ($w_{a}=0.3$) and then 20% for the stress at yield ($w_{\sigma \;}=0.2$), completely neglecting the strain values as an essential property for such an application ($w_{\varepsilon \;}=0$). This scheme is most suitable for TP applications, since the stiffness and the toughness are of the highest importance for TP products due to the stackability and durability requirements [48,49]. Therefore, this set of weights was chosen for the calculation of ${\mathrm{RQ}}^{\mathrm{mech}}$. By substituting the individual scores derived from the scoring functions of each property along with their assigned weights in Equation (8), a value of 0.3 is obtained for ${\mathrm{RQ}}^{\mathrm{mech}}$. Consequently, the same value is given to the factor $\alpha^{\mathrm{rec}:\mathrm{vir}}$, since it is determined as the minimum value of the two RQ factors (Equation (8)). Therefore, the technical substitutability of rPE-HD produced within this case study for TP applications is 0.3.

## 4. Discussion

This study provides a demonstration of an open-loop mechanical recycling process of a specific waste stream. One of the aims of this study was to track the journey of this waste stream throughout the whole recycling process and shed light on the possible changes in the materials. Additionally, the study was also an attempt to collect the essential processing and material data in the form of a digital product passport (DPP) and make them accessible to everyone [24]. Due to the increasing volume of bottled beverages and the limited potential for their recycling routes [15], the target waste stream was defined as PE-HD beverage bottle cap materials. The literature offers multiple studies that have investigated potential uses for plastic bottles and their components. Some of these studies favor the use of plastic bottles along with their caps in construction applications [79,80,81]. Others, on the other hand, proposed the usage of the post-consumer bottle caps as a core material in aluminum–plastic composite sandwich structures [19]. Mechanical recycling schemes of plastic bottles made of PET have evolved profoundly [16]. However, there is still room for development when it comes to their components, such as the caps.

The extensive material evaluation presented in this study revealed several aspects that need to be considered when recycling a certain waste stream. The compositional analysis of the defined input stream that was collected via two different collection strategies (informal and formal) enabled the assessment of these collection methods based on the technical properties of the input materials. It was found that the informal collection strategy resulted in a stream with a higher purity. This is because of the relatively higher PO cross-contamination, i.e., traces of PP in the PE-HD cap materials, which was detected by DSC measurements (Figure 7) in the formally collected stream and also by mixing these two streams in the rPE-HD recyclate. This can be attributed to the intrinsic motivation of the pupils and students to collect and separate the PE-HD caps according to the provided instructions. Nevertheless, PO cross-contamination is already a common phenomenon in the recycling sector of post-consumer plastics [82]. This phenomenon has been extensively studied through analytical research on waste materials [3,11,59,73,83,84,85] as well as on the resulting recyclates [3,73,86]. Despite the similarities in their chemical structures, PE and PP are immiscible polymers that are not supposed to be mixed [87,88]. Mixing them leads to the formation of a heterogeneous morphology that is characterized by phase separation, which in turn dramatically affects the material properties [73,88,89].

The property profile of the input streams, and subsequently the recyclate and the frisbee, were clearly influenced by the presence of the PP fraction in the material. While it is difficult to make a concrete statement that the MFR of Input-B was higher than that of Input-A due to the PO cross-contamination, the mechanical properties of the investigated materials were certainly affected by it. The structure–property relationships of both virgin and recycled PE/PP were elaborately investigated in multiple research studies. It was found that the presence of PP in PE, even at low concentrations, results in an increase in the material’s stiffness and strength and ultimately leads to a reduction in the strain at break values and toughness [72,73,75,78]. A comparison of these values between the two input streams in this study, as shown in Table 4, indicates a clear agreement with the existing literature. This confirms that the larger PP content in Input-B resulted in a slightly higher tensile modulus and stress at yield values and lower strain at break and NIS values compared to those of Input-A. The regranulation and injection-molding processes resulted in an improvement in the mechanical performance of both the recyclate rPE-HD and the final product, specifically in terms of tensile modulus and strength (i.e., stress at yield). Obviously, this can be attributed to the enhanced homogenization and removal of contaminants achieved by the recycling extruder during the regranulation process. However, the cross-contamination by PP, which was transferred to the resulting products, could possibly have contributed to the increase in these properties. Mechanical properties of POs are highly dependent on their degradation and crystallization behaviors. In the mechanical recycling process of plastics, both phenomena occur [63]. According to the literature, when processing PP the degradation mechanisms are primarily governed by chain scission causing a reduction in the molecular weight of the material, which leads to increasing the degree of crystallinity [69]. This increase in the degree of crystallinity corresponds to an increase in the tensile modulus and stress at yield, while the strain at break sinks [70]. This is due to the fact that in the crystalline phase the intermolecular bonds are much stronger than those in the amorphous phase, which hinders the movement of the macromolecules, thus leading to higher material’s stiffness and strength [70,90]. As a result, it can be assumed that the PP fraction in the input streams, and consequently in the recyclate, contributed to the higher tensile modulus and strength of the recyclate and subsequently the product.

Furthermore, one of the objectives of the study was to address the importance of social factors in the field of the circular economy of plastics and promote awareness of the beneficial use of plastics and plastic waste amongst young students. Therefore, the frisbee was selected as the pilot product because of its socio-technological suitability. The social aspect was accomplished by involving over a hundred classes from 19 different schools in the collection step of PE-HD beverage bottle caps. This approach intrinsically motivated the students to collect the targeted waste stream as instructed because they were given the opportunity to observe the transformation of their waste into a new product, to which they could relate. Moreover, the technological aspect of such a product lies in its resemblance of certain injection-molding applications where processability, mechanical properties, durability, and dimensional stability are of primary importance in comparison to other material characteristics. Transport packaging products, such as crates and boxes, were selected as potential applications where the resulting recyclate can be used. The assessment was based on the available market data derived from the TDSs of virgin grades and the substitution potential concept proposed by Demets et al. [47]. In their proposal, the substitutability factor is modified to take into consideration both the processability of the material as well as its technical functionality, which is described by its mechanical properties. The resulting recyclate, rPE-HD, returned scores of 0.5 and 0.3 in terms of its processability and its functional quality, respectively. Although the material is sufficiently processable by compatible technology, and it can deliver excellent toughness and strain at yield values, its tensile modulus and stress at yield fall outside of the optimal range. This means that the investigated recyclate is only 30% suitable for a full replacement of the available virgin grades. Nevertheless, in case of plastics recycling, for most applications except for PET bottles, it is known that a full substitution is not yet possible because of the inadequate quality of the recycled plastics [56]. Evidently, the tensile modulus and the stress at yield are below the minimal threshold of the prospect application by a just small margin. Hence, a partial replacement can be achieved through the implementation of a suitable modification technique that can enhance these specific properties without damaging the others [91,92]. Possible modifying methods include additivation of inorganic fillers or blending with virgin grades or even with other recyclates that have superior properties [93,94]. Nevertheless, this topic requires further consideration and a comprehensive analysis; thus, it cannot be sufficiently discussed in this section since property modification was not part of the current study. Additionally, in future studies, the economic applicability of such an open-loop recycling process including a separate collection of beverage bottle caps should also be considered and evaluated properly.

## 5. Conclusions

This study sheds light on the recycling potential of post-consumer PE-HD beverage bottle caps, which are mostly neglected due to the limited recycling paths for such a waste stream. A closed-loop recycling system is still not feasible in the case of this waste stream due to the demanding regulations and laws of food-contact grades. However, an open-loop recycling system to reintroduce the material into new products is always possible. The study revealed a cross-contamination, most likely due to poor separation efficiency, of the targeted materials, particularly in the formal collection step. The presence of the PP fraction in the material resulted in an increase in the tensile modulus and strength and a decrease in the toughness and strain at break. Furthermore, degradation and crystallization mechanisms of the PP fraction, represented by the increase in the melt enthalpy as well as a further increase in the material stiffness, were also observed in the properties of the recyclate after processing. These results are in agreement with the structure–property relationships that are reported in previous studies in the literature. Subsequently, all material and processing data were documented and stored in a digital product pass that maps out the property profile of the designated pilot product (i.e., the frisbee) throughout the entire value chain and makes its information accessible to everyone. This was an attempt to emphasize the importance of digitalization tools as facilitators for the transition to a CE for plastics. Finally, the technical functionality of the recyclate produced within the framework of this study was evaluated based on the market information to determine if it can be used for transport packaging applications. It was found that the potential for a full replacement is not possible. However, the implementation of certain modification methods might enhance the material properties and thus its substitution potential.

## Figures and Tables

**Table 1:** Summary of the relevant information of the investigated material states including their designation, source, processing technology, and form.

Designation	Source	Processing Technology	Form
Input-A	Schools (informal)	Shredding	Flakes
Input-B	Collection centers (formal)	Shredding	Flakes
rPE-HD	-	Regranulation	Pellets
Frisbee	-	Injection molding	Product

**Table 2:** Summary of the applied test methods on the materials with the designation of the different material states.

	Test Conditions			Input		Output1	Output2
Test Method\Material	Standard Method		No. of Measurements	Informal (A)	Formal (B)	Recyclate (rPE-HD)	Product (Frisbee)
	Material Processability						
Flake size distribution (FSD)	ASTM D1921		1, ca. 1 kg	X	X		
Apparent density (AD)	ASTM D1895, method C		3, 60.0 ± 0.2 g	X	X	X	
Melt flow rate (MFR)	ISO 01133-1, method B		3, ca. 4 g	X	X	X	X
	Thermal Analysis						
Ash content (AC)	ISO 3451-1, method A		3, ca. 4 g	X	X	X	X
Differential scanning calorimetry (DSC)	ISO 11357-1 and -3		3, 8 ± 1 mg	X	X	X	X
Oxidation induction temperature (OIT)	ISO 11357-1 and -6		3, 8 ± 1 mg	X	X	X	X
	Mechanical Tests						
Tensile tests	ISO 527-1 and -3		10 specimens, MPS and type 5	X	X	X	X
Charpy notched impact tests	ISO 179-1	−20 °C	10 specimens, type 1	X	X	X	
		23 °C					

**Table 3:** Summary of the melting temperature and enthalpies of the present peaks on the thermogram of the investigated materials generated by DSC measurements (2nd heating).

Material	Melting Temperature, T_m_ [°C]				Melting Enthalpy, ∆H [J/g]			
	PE-HD		PP		PE-HD		PP	
Input-A	131.4	±0.0	160.4	±0.4	190.3	±5.8	2.1	±0.4
Input-B	131.0	±0.1	159.9	±0.1	171.6	±3.4	7.7	±0.4
rPE-HD	131.5	±0.1	160.4	±0.1	180.0	±1.1	6.6	±0.2
Frisbee	131.2	±0.2	160.2	±0.1	165.9	±0.6	9.6	±0.1

**Table 4:** Summary of the mean values together with the standard deviation of the mechanical properties of the investigated materials generated by tensile and Charpy notched impact tests.

Property		Input-A		Input-B		rPE-HD		Frisbee ^1^	
Tensile modulus (MPa)		810.4	±21.9	823.9	± 9.6	850.1	±9.8	1186.0	±39.5
Stress at yield (MPa)		21.1	±0.4	21.2	±0.1	21.5	±0.2	25.4	±0.6
Strain at yield (%)		11.1	±0.3	11.0	±0.2	10.7	±0.2	7.6	±0.3
Strain at break (%)		422.8	±53.1	211.8	±78.8	338.4	±91.5	114.8	±74.1
Charpy notched impact strength (kJ/m^2^)	23 °C	8.3	±0.1	7.6	±0.4	7.0	±0.1	-	-
	−20 °C	3.8	±0.0	3.4	±0.5	3.7	±0.0	-	-

^1^ Tensile properties of type 5 specimens cut from the flat surface of the frisbees.

## Data Availability

The data presented in this study are available on request from the corresponding authors.
